# Characterization of 9-Nitrocamptothecin Liposomes: Anticancer Properties and Mechanisms on Hepatocellular Carcinoma *In Vitro and In Vivo*

**DOI:** 10.1371/journal.pone.0021064

**Published:** 2011-06-09

**Authors:** Shunzhen Zheng, Shuang Chang, Jinli Lu, Zhihui Chen, Li Xie, Yu Nie, Bin He, Shengquan Zou, Zhongwei Gu

**Affiliations:** 1 Department of General Surgery, Tongji Hospital, Tongji Medical College of Huazhong University of Science and Technology, Wuhan, People's Republic of China; 2 National Engineering Research Center for Biomaterials, Sichuan University, Chengdu, People's Republic of China; Univesity of Texas Southwestern Medical Center at Dallas, United States of America

## Abstract

**Background:**

Hepatocellular carcinoma (HCC) is the third most common cause of cancer related mortality worldwide. 9-Nitrocamptothecin (9NC) is a potent topoisomerase-I inhibitor with strong anticancer effect. To increase the solubility and stability, we synthesized a novel 9NC loaded liposomes (9NC-LP) via incorporating 9NC into liposomes. In the present study, we determined the effects of 9NC and 9NC-LP on *in vitro* and *in vivo*, and the underlying mechanisms.

**Methodology/Principal Findings:**

We first analyzed the characteristics of 9NC-LP. Then we compared the effects of 9NC and 9NC-LP on the proliferation and apoptosis of HepG2, Bel-7402, Hep3B and L02 cells *in vitro*. We also investigated their anticancer properties in nude mice bearing HCC xenograft *in vivo*. 9NC-LP has a uniform size (around 190 nm) and zeta potential (∼−11 mV), and exhibited a steady sustained-release pattern profile *in vitro*. Both 9NC and 9NC-LP could cause cell cycle arrest and apoptosis in a dose-dependent and p53-dependent manner. However, this effect was not ubiquitous in all cell lines. Exposure to 9NC-LP led to increased expression of p53, p21, p27, Bax, caspase-3, caspase-8, caspase-9 and apoptosis-inducing factor, mitochondrion-associated 1 and decreased expression of Bcl-2, cyclin E, cyclin A, Cdk2 and cyclin D1. Furthermore, 9NC-LP exhibited a more potent antiproliferative effect and less side effects *in vivo*. Western blot analysis of the xenograft tumors in nude mice showed similar changes in protein expression *in vivo*.

**Conclusions/Significance:**

In conclusion, 9NC and 9NC-LP can inhibit HCC growth via cell cycle arrest and induction of apoptosis. 9NC-LP has a more potent anti-tumor effect and fewer side effects *in vivo*, which means it is a promising reagent for cancer therapy via intravenous administration.

## Introduction

9-Nitrocamptothecin (9NC) is a potent topoisomerase-I inhibitor, pharmacological studies disclosed that the antitumor activity of 9NC was superior to that of camptothecin (CPT) in human tumors xenografted in nude mice [1]. The antitumor activity of 9NC closely depended on its structure: the lactone form of 9NC was important to its antitumor activity. However, the maintenance of the lactone form is a challenge for the clinical application of 9NC. The applications of 9NC were also limited due to its poor solubility, instability and low oral bioavailability [2], [3]. The poor water solubility would induce opsonization and cause rapid clearance in blood circulation after intravenous injection [4]. Recently, several novel drug delivery systems (DDS) have been used in 9NC tried to address problems mentioned above, among the many DDS available, both micelles and liposomes have gained the most attention [5], [6].

Hepatocellular carcinoma (HCC) is the third most common cause of cancer related mortality worldwide [7]. The incidence of HCC has been increasing dramatically in the past decades and it is expected to continue to increase for the next two decades [8], [9]. In 2002, the incidence of HCC in Chinese male and female was about 250,000 and 90,000, respectively (International Agency for Research on Cancer: http://www-dep.iarc.fr/). In the United States, primary liver cancer is projected to contribute more than 21,000 new cases and cause 18,000 deaths during 2008. HCC is characteristic of progressive development and rapid metastasis. Systemic therapy with cytotoxic agents has historically been of marginal benefit. Exploration of more potential chemotherapeutic agents is gaining increased attention [10].

To improve the stability and water solubility of 9NC, we constructed a novel liposome system containing 9NC via entrapping 9NC in liposomes with film-ultrasonic method. We then compared the anticancer properties of 9NC and 9NC loaded liposomes (9NC-LP) *in vitro* and *in vivo*. We also determined the effects of 9NC and 9NC-LP on the expression of several proliferation and apoptosis related molecules to investigate the underlying mechanisms.

## Results

### Preparation of 9NC loaded liposomes and its releasing characterization

**Figure 1:**
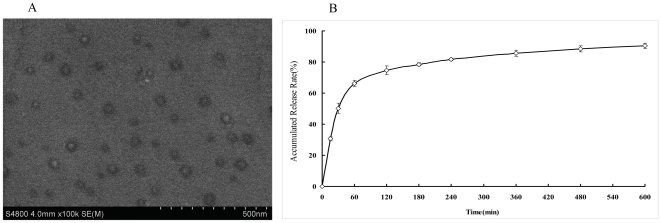
SEM image and *in vitro* release profile of 9NC loaded liposomes. *(A)* SEM image of 9NC loaded liposomes. *(B) In vitro* release profile of 9NC from liposomes in pH 7.4-PBS at 37±1°C by the dialysis method. The results are expressed as mean±SD (n = 3).

The average size of 9NC loaded liposomes before and after lyophilization was 160∼200 nm, with a very narrow size distribution (Table 1). Encapsulation of 9NC slightly increased the size of particles from 160 nm to 190 nm. There was no detectable difference of the zeta potential (∼−11 mV) and encapsulation efficiency (4.5%) of liposomes after lyophilization, which confirmed no degradation or aggregation occurred during the lyophilization and storage. As showed in SEM (Fig. 1A), 9NC encapsulated liposomes was of regular spherical shape, while with smaller size (∼50 nm) than that detected by photon correlation spectroscopy. It should be noted that the difference in the detected sizes is due to the fact that photon correlation spectroscopy gives the hydrodynamic diameter, while SEM shows the shape of dried particles. The *in vitro* release of the 9NC loaded liposomes was presented in Fig. 1B. The release time of the liposomes could last for 600 minutes and the burst release is not obvious, which implied that the hydrophobic 9NC was successfully encapsulated in the liposomes.

**Table 1:** Characteristics of 9NC encapsulated liposomes.

	Size(nm)	Zeta potential(mV)	Encapsulation efficiency
Before lyophilization	162.4±9.4	−11.5±9.5	4.6±0.2%
After lyophilization	199.6±9.8	−11.5±7.1	4.5±3.1%

### Screening for *in vitro* growth inhibition

The compounds were first tested for their *in vitro* cytotoxicity via MTT assay. As Table 2 shows HepG2 cell line consistently was more sensitive in both 9NC and 9NC-LP, while Hep3B was resistant to both drugs.

**Table 2:** Cell growth inhibitory activity of 9NC and 9NC-LP in human cancer and normal cells.

Incubation time	IC50(µmol/L)^a^							
	HepG2		Bel-7402		Hep 3B		L02	
	9NC	9NC-LP	9NC	9NC-LP	9NC	9NC-LP	9NC	9NC-LP
10 h	29.39±1.26	37.56±1.78	34.05±0.95	46.8±1.39	346.36±14.96	367.81±20.33	39.94±0.93	51.43±2.45
24 h	16.33±0.72	18.81±0.89	17.46±1.03	18.00±0.94	275.15±11.58	290.41±18.71	17.87±0.63	23.90±0.36
48 h	6.28±0.12	1.53±0.08	6.83±0.29	1.61±0.04	17.19±1.34	34.39±1.52	29.14±0.52	14.52±0.48
72 h	0.17±0.01	0.02±0.00	0.41±0.01	0.29±0.01	7.28±0.43	18.56±0.52	12.58±0.08	2.66±0.05

a IC50, the concentration that inhibits cell growth by 50%.

### Cell Proliferation assay

**Figure 2:**
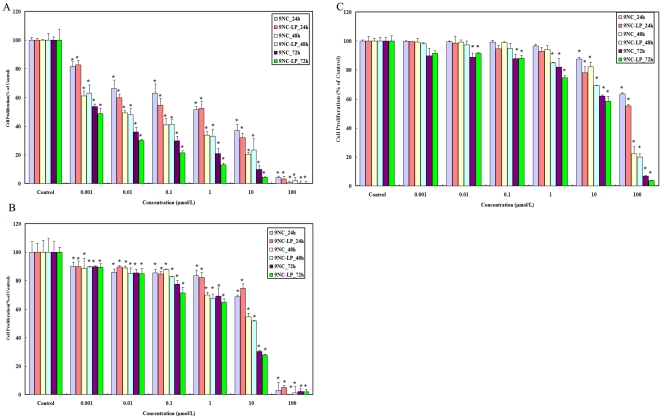
Cell proliferation inhibitory effects of 9NC and 9NC-LP on HepG2, L02 and Hep3B cells *in vitro*. Cell proliferation of HepG2*(A)*, L02*(B)* and Hep3B*(C)* cells was measured after exposure to compounds for 24–72 h. All assays were done in triplicate. The proliferation index is in comparison with untreated cells (*P<0.01, ^<$>\raster(60%)="rg1"<$>^P<0.05 vs. control) (There no significant differences between cells treated with 4‰DMSO, Liposomes (free) and untreated cells. Data not shown).

To further determine the effects of 9NC and 9NC-LP on cell proliferation, HepG2, Hep3B and L02 were subject to BrdU proliferation assay. The proliferation of all cell lines was suppressed after the treatment with 9NC and 9NC-LP. The antiproliferative effects of both drugs were dose- and time- dependent. As showed in Fig. 2 HepG2 cell line was more sensitive in both 9NC and 9NC-LP. The results are similar to that of the MTT assay.

### Effects of the compounds on cell cycle arrest

**Figure 3:**
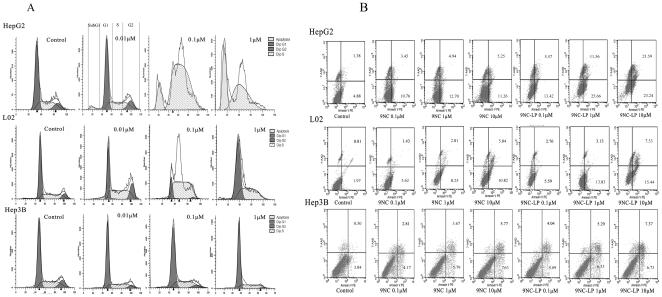
Cell cycle arrest and apoptosis-induced effects of 9NC and 9NC-LP on HepG2, L02 and Hep3B cells *in vitro*. For the cell cycle analysis, cells were seeded at 2×10^5^ to 3×10^5^ per well in six-well paltes in triplicates and incubated with compounds. After treated for 24 h, 48 h and 72 h, cells were fixed, permeabilized, stained with PI and analyzed by flow cytometry. Results showed cells were incubated with 0, 0.01, 0.1, 1 µmol/L 9NC-LP for 24 h*(A)*. Apoptosis of HepG2, L02 and Hep3B cells*(B)* was measured by flow cytometry after stained with AnnexinV-PE and 7-AAD (Concentration: 0, 0.1, 1, 10 µmol/L). This experiment was done in triplicate and representative diagrams are shown (There no significant differences between cells treated with 4‰DMSO, Liposomes (free) and untreated cells. Data not shown).

9NC and 9NC-LP could induce apoptosis (increased Sub G1 peak) (p<0.05) and cause cell cycle arrest (p<0.05) of the HCC cell lines. The effect is dose- and time-dependent in all tested cell lines. Cell cycle phase is alternation according to the compound concentration and incubation time in all test cell lines. S phase delay was observed after exposure for 24 h and G2/M phase delay was observed after exposure for 72 h in all tested cell lines (Fig. 3A, Table S1). Both compounds caused more significant cell cycle arrest in the HepG2 cells than in other cell lines. Almost all of the HepG2 and Bel-7402 cells arrest in S phase when compounds concentrations of both compounds were over 0.05 µmol/L while L02 and Hep3B cells were more resistant.

### Induction of apoptosis

To confirm the effects of 9NC and 9NC-LP on cell apoptosis, we next evaluated apoptosis induced by compounds on HepG2, Hep3B and L02 via by *FACSC*alibur flow cytometry after the cells stained with Annexin V-PE/7-AAD. As illustrated in Fig. 3B, both the compounds induced apoptosis in a dose-dependent manner in all tested cell lines. Similarly, the Hep3B cell line was less sensitive, whereas HepG2 cell line was more sensitive. The results were in accordance with the Sub G1 assay.

### *In vivo* activity against human hepatocellular carcinoma xenograft tumors

**Figure 4:**
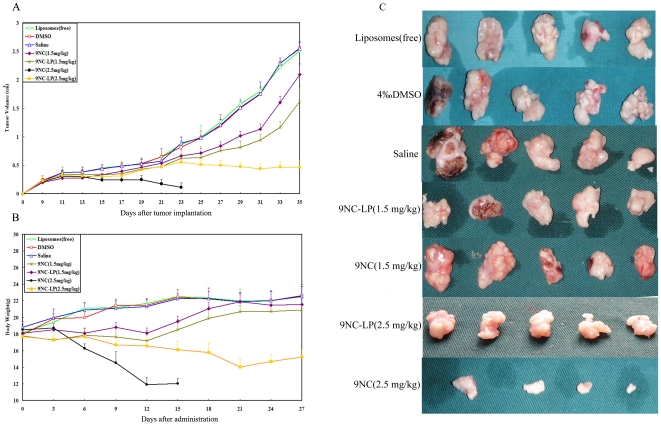
*In vivo* effects of 9NC and 9NC-LP given to nude mice bearing HepG2 xenograft tumors. Compounds were given i.v. at doses of 1.5 mg/kg/d or 2.5 mg/kg/d on a 5/2/5 (5 days on, 2 days off and 5 days on) schedule for 3 weeks. Tumor size was determined every two day after tumor implantation. Tumor volume (cm^3^) = (*w* ^2^×*l*)/2, *w* = width, *l* = length of the tumor *(A)*. (Values presents as means ± SD. Liposomes (free): p>0.05; DMSO: p>0.05; 9NC (1.5 mg/kg): p<0.05; 9NC-LP (1.5 mg/kg): p<0.05; 9NC-LP (2.5 mg/kg): p<0.01; vs. Saline respectively. The data of 9NC (2.5 mg/kg) was removed from statistics for more than half animals were killed during the study). Animals were also monitored for changes in body weight every three days after administration as a surrogate marker for toxicity *(B)*. At the end of the experiment, representative tumors were removed and photographed *(C)*.

To determine the antitumor activity of 9NC and 9NC-LP *in vivo*, we evaluated their effect in a nude mice xenograft model of HepG2 cell line. Both reagents were administered at 1.5 mg/kg/d or 2.5 mg/kg/d on a 5/2/5 (5 days on, 2 days off and 5 days on) schedule for 3 weeks. The higher dose of (2.5 mg/kg/d)9NC-LP inhibited tumor growth by about 87.02% on day 25, and the lower dose (1.5 mg/kg/d) also inhibited tumor growth significantly (41.66%; P<0.01) (Supplementary Tables 2). No significant loss of body weight was observed when the mice were treated with 9NC-LP at the dose of 1.5 mg/kg/d, while significant weight loss was detected at the dose of 2.5 mg/kg/d. However, there was significant weight loss in mice treated with 9NC of both dose groups. Furthermore, over half of the animals were killed by 9NC at day 14 after administration of 2.5 mg/kg/d doses group. Body weight loss, diarrhea, hemorhaging cystitis (hematuria) and treatment-related death were observed in 9NC group while its incidence is lower in 9NC-LP group (Fig. 4, Table 3, Table S2).

**Table 3:** Toxicity of 9NC and 9NC-LP in human hepatocellular carcinoma xenograft tumor model.

Treatments	body weight loss	diarrhea	hemorhaging cystitis(hematuria)	treatment-related death
Liposomes(free)	0/10	0/10	0/10	0/10
4‰DMSO	0/10	0/10	0/10	0/10
Saline	0/10	0/10	0/10	0/10
9NC 1.5 mg/kg	6/10	4/10	3/10	1/10
9NC-LP 1.5 mg/kg	2/10	0/10	0/10	0/10
9NC^a^ 2.5 mg/kg	10/10	10/10	10/10	6/10
9NC-LP 2.5 mg/kg	10/10	4/10	1/10	0/10

All animals were monitored from day 1 to day 27 after administration, the animal was defined as positive once item appearance. The values presented the total number of item positive animals in the 10 mice in each group.

a In this group, 6 mice were dead caused by treatment on day 14 after administration, the others were sacrificed and tumors were removed for the protein expression analysis on the same day.

### 9NC and 9NC-LP affect expressions of proteins associated with cell cycle progression and apoptosis

**Figure 5:**
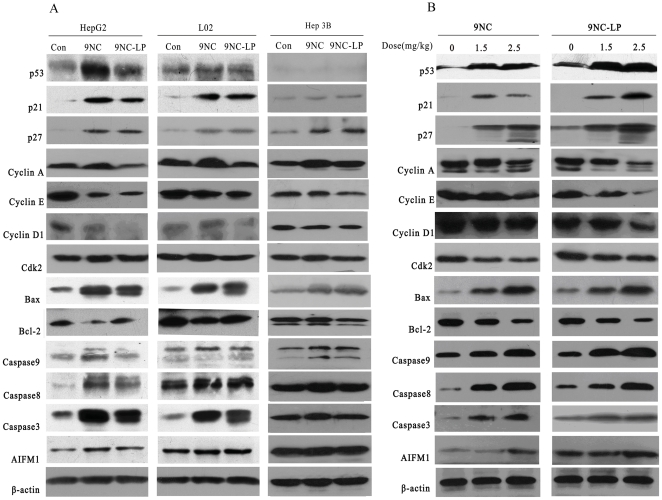
Effects of drugs on the expression of proteins related to cell cycle progression and apoptosis in human hepatocellular carcinoma cell line and normal liver cell line. *In vitro*, HepG2, L02 and Hep3B cells were exposed to the compound (1 µmol/L) for 24 h; then target proteins were examined by western blotting*(A)*. *In vivo*, compounds were given i.v. at doses of 1.5 mg/kg/d or 2.5 mg/kg/d on a 5/2/5 (5 days on, 2 days off and 5 days on) schedule for 3 weeks. The expression of proteins from tumor homogenates was analyzed by western blotting *(B)*.

We then investigated the possible mechanisms responsible for the effects of 9NC and 9NC-LP by evaluating its effects on the expression level of various proteins involved in regulating cell cycle progression and apoptosis. In both HepG2 and L02 compounds activated p53; increased the expressions of p21, p27, Bax, Caspase-3, Caspase-8, Caspase-9 and apoptosis-inducing factor, mitochondrion associated 1 (AIFM1); decreased the expressions of Bcl-2, cyclin A, CDK2, cyclin E and cyclin D1. In Hep3B cell, no expression of p53 was detected and effects of compounds on cell cycle-related and apoptosis-related proteins expression is less than HepG2 and L02 cells. Interestingly, the patterns of protein expression in the treated xenograft tumors were similar with those observed *in vitro* (Fig. 5).

## Discussion

This study was designed to evaluate the anticancer activities of 9NC and 9NC-LP *in vitro* and *in vivo* human cancer models. We have shown several important points: (a) the remarkable anticancer activity of 9NC and 9NC-LP are dose- and cell type- dependent; although cell cycle arrest is the major mechanism responsible for the cytotoxicity of the compounds, apoptosis was also observed; (b) the compound upregulated p53 and regulated the expression of cell cycle-related, apoptosis-related proteins, including p21, p27, Cdk2, Cyclin A, Cyclin E, Cyclin D1, Bcl-2, Bax, Caspase-3, Caspase-8, Caspase-9 and AIFM1; (c) both 9NC and 9NC-LP inhibited the growth of xenograft tumors in mice in a dose-dependent manner, nevertheless 9NC-LP show an apparently more potent antitumor effect and less side effect; and (d) similar changes in protein expression were observed *in vitro* and *in vivo* after exposure to compounds.

It is generally accepted that tumors have ‘leaky vasculature’ due to endothelial gaps and irregular basement membranes [11]. Interendothelial gap defects increase vascular permeability in tumors, leaky blood vessels would allow liposomes up to 400 nm to passively accumulate the tumors at these sites [12], this property is called the enhanced permeability and retention (EPR) effect [13], [14]. Our data shown 9NC-LP has a uniform size and less negative potential. The release profile exhibited a steady sustained-release pattern without obvious burst release. These characteristics imply 9NC-LP suitable for intravenous administration.

Rapid release of drug from nanocarriers is still a challenge of lipid-based vesicles; about 90% 9NC was released from liposomes after 10 h *in vitro* in our study. Though there no obvious burst release was observed, the rapid release means the formulation needs more modifications to prolong its release time.

The present data showed that anti-proliferative effects were cell type-dependent. In general, for both drugs, the order of sensitivity was HepG2>Bel-7402>L02>Hep3B. The MTT assay results indicated 9NC showed a stronger inhibitory effect after cells were incubated for 10 h while 9NC-LP showed a stronger inhibitory effect after cells were incubated for 48 h and 72 h; this can be explained by the *in vitro* release profile of 9NC-LP, because 9NC had not been released from liposomes completely after 10 h. Cell cycle analysis showed that both drugs caused cell cycle arrest in all cell lines. The ratio of S and G2/M phase cell increased sequentially while the concentration and incubation time of the reagents increased. This is prominent in HepG2 and Bel-7402 cell. The ratio of Sub G1 population increased along with compound concentration and incubation time increasing in all test cell lines.

The results of cell proliferation assay and detection of apoptosis are in accordance with MTT assay and Sub G1 method. The present data showed that both the most common causes of cell growth inhibition-cell cycle arrest and apoptosis [15] - were involved in the anticancer activity of 9NC and 9NC-LP.

As an inhibitor of topoisomerase I, 9NC can attach to DNA and form a “dead-end” complex which prevents topoisomerase-I from resealing the DNA strand after the incision step [16]. This “dead-end” complex causes DNA damage which can also cause growth arrest and apoptosis. In this work, S and G2/M phase delay was prominent, precise mechanism(s) must be extremely complicated. Moreover, p53 plays a key role in the cell cycle delay; its effect is involved in G1, S and G2/M phase arrest [17]. In mammalian cells, the p53 tumor suppressor is usually responsible for the growth arrest and apoptosis caused by DNA damage. Cdks associate with cyclins to form heterodimers that are sequentially activated during the cell cycle [18]. In normal cell cycles, Cdk2 pairs with E- and A-type cyclins during S phase. The the Cip/Kip family (such as p21CIP1 [19] p27KIP1 [20]) can inhibit activity of cyclin-Cdk heterodimers and cause phase-specific cell cycle arrest [21]. Activated p53 can induce the expression of p21Cip1 which can inhibit the S phase promoting factor (cyclin A-Cdk2 or cyclin E-Cdk2 kinase complexes) , thereby causing a damaged cell to arrest in S phase [22], [23]. This is supported by the cell cycle profile and activation of cell cycle-related proteins observed by western blotting.

Apoptosis is also an important mechanism which is supported by presence of Sub G1 peak in flow cytometry results and up-regulation of apoptosis-related proteins in western blotting results. The mammalian p53 can upregulate the expression of several proapoptotic Bcl2-homology-domain proteins, such as Bax, which can activate apoptosis [24]. In this study, mitochondrial apoptosis pathway has also been observed which was supported by increase of caspase-9, AIFM1, Bax and decrease of Bcl-2. Of note, apoptosis was observed at higher concentration and longer incubation time than cell cycle delay which indicate apoptosis appeared relative late.

In our study, the p53-deficient cell Hep3B was more resistant to drugs. This may be explained by the p53 activity. Usually, p53 have a very short half-life, its half-life can be extended by DNA damage. Accumulation of p53 protein results in cell cycle arrest and cell apoptosis. However, in p53-deficient cells, cell division is not arrested despite the damage to DNA [25].

After evaluating the effects of the compounds on proliferation, apoptosis, and cell cycle progression, we concluded that cell cycle arrest was the main mechanism that 9NC exert its effects. The modulation of cell-cycle progression in cancer is considered an effective strategy to inhibit tumor growth [26]. The assumption is based on a molecular analysis of human cancer, wherein cell cycle regulators are frequently de-regulated in most of common malignancies [27], [28].

After our observation that 9NC and 9NC-LP exerted potent effects against hepatocellular cancer cells *in vitro*, we initiated a study to determine whether the compound could inhibit tumor growth in a HepG2 xenograft model *in vivo*. As Fig. 4 indicates 9NC-LP shows an outstanding performance *in vivo*. A dose of 1.5 mg/kg/d and 2.5 mg/kg/d 9NC-LP for 3 weeks led to 41.66% and 87.02% tumor growth inhibition respectively, without any drug-related death. Nevertheless, half of the animals were killed at day 14 after administration in 2.5 mg/kg/d doses 9NC group. Body weight loss, diarrhea, hemorhaging cystitis (hematuria) and treatment-related death were observed in 9NC group while its incidence is lower in 9NC-LP group. The present data showed that liposomes delivery systems could improve the 9NC antitumor activity while reduce the side effect. On the one hand, this may because 9NC existed in active lactone/inactive carboxylate equilibrium, since the carboxylate form limited the antitumor activity and increased the toxic side effects to normal tissues [29]. Encapsulation of 9NC help to maintain the lactone that can improve therapy effect and decrease side effects [30], [31].On the other hand, the effect of 9NC-LP can be explained by the following reasons; due to the small size, liposomes readily extravasate from circulation through vascular gaps or defects [32], which have been reported to be ∼200 nm or greater [12], [33]. 9NC-LP retention within these sites is generally high due to the poor lymphatic drainage observed within tumors [34]. Furthermore, their lower size limit in diameter ensures that these vehicles do not randomly penetrate normal vessel walls. As a result, 9NC-LP minimizes the undesirable side effects which can occur using conventional drugs [35], [36].

Liposomal formulations of anticancer agents have already been approved for human use. And the clinical success has made them very popular drug carriers for various chemotherapeutics. For example, the clinically approved drugs DaunoXome and Doxil are liposomal formulations that encapsulate the commonly used chemotherapeutic agents daunorubicin and doxorubicin respectively [5].

Liposomes are composed of a phospholipid bilayer which entirely surrounds an internal aqueous core. The liposomes bilayer can be composed of either synthetic or natural phospholipids. Liposomes are larger than micelles and therefore have the ability to deliver greater amounts of the chemotherapeutic agent to the tumor site while minimizing the risk associated with premature leakage. In addition, liposomes also have the ability to accommodate both hydrophilic as well as hydrophobic drugs, either in the internal aqueous core or in the lipid bilayer, respectively [37]. Otherwise, liposomes can easily be manipulated by adding additional molecules to the outer surface of the lipid bilayer.This offers a significant advantage over other carriers that require much more controlled synthesis steps and additional chemical modifications [38]. When compared to conventional (unencapsulated) drugs, liposomal treatment has been shown to dramatically reduce some of the traditional side effects associated with chemotherapy [5]. It's noteworthy, liposomes was found own the potential to combat the increasing problem of multidrug resistance (MDR) acquired by cancers, which drastically reduces chemotherapeutic efficacy [39].

Liposomes have demonstrated multiple advantages as drug delivery vehicles. However, lipid-based vesicles also pose several challenges such as instability in the bloodstream, poor solubility of many drugs in the lipid/surfactant solution, and a rapid, burst release of drug.

To date, no specific *in vivo* study has compared the efficacy of liposomes to that of other nanoparticle delivery systems; therefore, we cannot comment on the relative efficacy of liposomes [39].

In conclusion, we have incorporated camptothecin analogues into liposomes and synthesized a new 9NC formulation which can be administered intravenously. 9NC-LP can inhibit HCC growth via cell cycle arrest and apoptosis induction. Activation of p53 may be one of the important regulators in 9NC-LP caused cell death. More importantly, 9NC-LP has more potent effects and fewer side effects *in vivo*. Further molecular, pharmacologic, and toxicological studies are needed to elucidate the underlying mechanisms and to determine the optimal dose. These studies are important for further preclinical and clinical development of this class of compounds.

## Materials and Methods

In this study, the animal use and care protocol was approved by the Institutional Animal Use and Care Committee of the Huazhong University of Science and Technology (Permit Number: 20090901).

### Chemicals and reagents

9-nitrocamptothecin (9NC) was purchased from Hangzhou Heta Pharm& Chem Co., Ltd. Cholesterol was from Aladdin reagent (Shanghai, China). Egg yolk lecithin PC-98(egg yolk phosphatidylcholine) was obtained from Q.P. Corporation Fine Chemical Division (Japan).All chemicals and solvents used were of the highest analytic grade available. Cell culture supplies and media, PBS, fetal bovine serum, and penicillin/streptomycin were obtained from HyClone Thermo Fisher Scientific Inc. Anti-human Caspase-8, Caspase-9, apoptosis-inducing factor, mitochondrion-associated 1(AIFM1) antibodies were from Proteintech Group, Inc. The anti-human p53, p21, p27, Caspase-3, Bcl-2, Bax, cyclin A, cyclin E, cyclin D1, cyclin-dependent kinase (Cdk)2 antibodies were from Santa Cruz Biotechnology, Inc. 9NC was dissolved in dimethyl sulfoxide (DMSO, Sigma-Aldrich, Germany) as a stock solution of 10 mg/mL and added to the cells in the indicated concentrations. 4‰ DMSO (the highest concentration used to dilute the 9NC in this study) and liposome without any 9NC were included in all assays as control group.

### Preparation and characterization of 9NC loaded liposomes

9NC loaded liposomes were prepared by classic film-ultrasonic method. Briefly, the lipid mixture (phosphatidylcholine: cholesterol = 2∶1, w/w) and 9NC (with drug to phosphatidylcholine = 1∶5, w/w) were dissolved in appropriate mixture solution of dichloromethane and methanol (3∶1, v/v), and a thin lipid film was formed by rotary evaporating at 40°C under reduced pressure. Resulted dried lipid film was vacuum-dried overnight to get rid of residual organic solvent. Then the film was hydrated in 8 mL of D-Hanks solution (0.01 M, pH 6.5) with 9NC. Subsequently, the suspension was sonicated in an ice bath to clarity. After preparation, unencapsulated free 9NC were separated by ultrafiltration, and the encapsulation efficiency was calculated according to previous study [40]. The mean particle size (z-average) of liposomes was measured by photon correlation spectroscopy (Nano ZS90; Malvern, UK). A 1∶100 dilution of the formulations was made using double-distilled water before the zeta potential measurement. Morphology of the liposomes was characterized by scan electron microscopy (SEM).

### Release and lyophilization of liposome

Release of camptothecin from the drug carrier systems was measured using dialysis method with a molecular weight cut-off of 10, 000 Da. The receptor medium consisted of isotonic phosphate buffer solution (137 mM NaCl, 3 mM KCl, 8 mM Na_2_HPO_4_, 1 mM KH2PO_4_, pH 7.4) containing 0.05% Tween 80, which was constantly stirred at 37±1°C in order to maintain sink conditions during the experiments [40]. At appropriate intervals, 1 mL aliquots of the receptor medium were withdrawn and immediately replaced with an equal volume of fresh buffer. The amount of drug released was determined by UV. A certain amount of D-trehalose was added into freshly prepared liposomes for freeze-dry in Eppendorf tubes with final concentration of 1.2%. Before lyphilization, solution was sterilized by filtering through 0.22 µmol/L membrane. The tubes were prefrozen at −80°C for 24 h, and placed into the drying chamber of the Freeze Dry System (FD-IC-50, Boyikang Experiment Device Company) for 24 h-drying performance. Physicochemical properties of lyophilized liposomes were investigated, including size, surface charge and entrapment efficiency.

### Cell culture

The HepG2, Bel-7402 and Hep3B human HCC cell lines and L02 normal liver cell line was involved in this study. HepG2 and L02 were obtained from the China Center for Type Culture Collection. Bel-7402 purchased from the cell bank of Chinese Academy of Science. Hep3B is a p53-deficient human HCC cell line and was obtained from American Type Culture Collection [41], [42]. HepG2 and L02 were routinely passaged in DMEM (High Glucose) Media while Bel-7402 was passaged in RPMI 1640 media. Hep3B was passaged in MEM media. All cell culture media contained 10% fetal bovine serum and 1% penicillin/streptomycin. Cultures were maintained at 37°C in the presence of 5% CO_2_.

### Cell survival assay

The effects of the test compounds on cell viability were determined with the use of the 3-(4, 5-dimethylthiazol-2-yl)-2, 5-diphenyltetrazolium bromide (MTT) assay. Cells were seeded in 96-well plates at 4×10^3^ to 5×10^3^ cells per well and exposed to different concentrations of the test compounds (0, 0.001, 0.01, 0.1, 1, 10, or 100 µmol/L). After incubation for 10 h, 24 h, 48 h and 72 h, 10 µL of the MTT solution (5 mg/mL; Sigma) were added into each well. The plates were incubated for further 4 h at 37°C. Thereafter, the supernatant was removed, and the formazan crystals were dissolved with 100 µL of DMSO. The absorbance at 490 nm was recorded with the use of a microplate reader (Molecular Devices).

### Cell proliferation assay

To further determine the effects of 9NC and 9NC-LP on cell proliferation, cell proliferation ELISA assay (BrdU (colorimetric); Roche Diagnostics) was used in this study. HepG2, Hep3B and L02 cells were seeded in 96-well plates at a density of 4×10^3^ to 5×10^3^ and incubated with 9NC and 9NC-LP (0, 0.001, 0.01, 0.1, 1, 10, or 100 µmol/L) for 24, 48 and 72 h. The determination of the cell proliferation was carried out according to the manufacturer's instruction manual. The mean absorbance of the untreated wells was defined as 100%; absorbance of the other wells was then related to this value.

### Cell cycle measurements and Sub G1 FACS assay

Cells (2×10^5^ to 3×10^5^) were exposed to the test compounds (0, 0.01, 0.025, 0.05, 0.1, 0.5, 1, or 10 µmol/L) and incubated for 24 h, 48 h and 72 h before analysis. The cells were harvested and fixed in 70% ethanol at −20°C overnight, followed by incubation with RNase and staining with propidium iodide (Sigma-Aldrich, Germany). The DNA content was determined by *FACSC*alibur flow cytometer (Becton Dickinson Biosciences, San Jose, CA).

### Detection of apoptosis

HepG2, Hep3B and L02 cells in early and late stages of apoptosis were detected using of an Annexin V-PE/7-AAD apoptosis detection kit from KeyGEN according to the manufacturer's protocol. 2×10^5^ to 3×10^5^ cells were exposed to the test compounds (0, 0.1, 1, 10 µmol/L) for 24 h before analysis. The cells were collected and washed with serum-free media, then resuspended in binding buffer followed by the addition of 5 µL of Annexin V-PE and 5 µL of 7-AAD. The samples were incubated in the dark and were analyzed with a Becton Dickinson FACSCalibur instrument. The cells that were positive for Annexin V- PE alone (early apoptosis), and Annexin V- PE and 7-AAD (late apoptosis) were counted.

### Human xenograft models and animal treatment

Female athymic pathogen-free nude mice (BALB/c, 3–4 wk) were purchased from Shanghai SLAC laboratory Animal Co., Ltd. (SLAC). To establish HepG2 human hepatocellular cancer xenograft tumors, HepG2 cells were harvested from monolayer cultures, washed twice with serum-free medium, resuspended, and injected s.c. (1×10^7^ cells; total volume, 0.2 mL) into the backs area of the mice. Ten days after implantation, mice were randomly sorted into treatment groups (10 mice in each group) and were administrated intravenously (i.v.) via tail vein with saline, 9NC-LP (1.5 mg/kg/d), 9NC-LP (2.5 mg/kg/d), 9NC (1.5 mg/kg/d) and 9NC (2.5 mg/kg/d). Mice were treated i.v. (0.2 mL/20 g body weight) with drugs on a 5/2/5 (5 days on, 2 days off and 5 days on) schedule. Tumor size was determined every two day by caliper measurement of two perpendicular diameters of the implant. Body weight was monitored every three days during the studies. All animals were monitored for activity, physical condition, body weight, and tumor growth. Animals were sacrificed on day 27 after administration; tumors were removed for the protein expression analysis.

### Western blot analysis

The protein levels in cell lysates and tissue homogenates were assessed with the use of methods described previously [43]. In the *in vitro* studies, cells were exposed to of 1 µmol/L 9NC and 9NC-LP. Western blot experiments were carried out by regular SDS-PAGE. Cell lysates with identical amounts of protein were fractionated and transferred to Immobilon™ PVDF Transfer Membranes (Millipore). The PVDF membrane was incubated in blocking buffer (TBS containing 0.1% Tween 20 and 5% nonfat milk) for 2 h at room temperature. Then the membrane was incubated with the appropriate primary antibody overnight at 4°C or 2 h at room temperature with gentle shaking. The membrane was washed and then incubated with goat anti-mouse/rabbit IgG-horseradish peroxidase-conjugated antibody (Santa Cruz Biotechnology, Inc.) for 1 h at room temperature. After repeating the washes in triplicate, the protein of interest was detected by enhanced chemiluminescence reagents from Pierce, Thermo Fisher Scientific Inc..

### Data and statistical analysis

Experimental data are expressed as mean ± standard deviation(SD), and the significance of differences was analyzed by Repeated Measures Analysis of Variance, one-way analysis of variance (ANOVA), Multi-factor Analysis of Variance and post hoc test or Student's t test as appropriate.

## Supporting Information

Table S1 Effects of 9NC and 9NC-LP on the cell cycle progression.
(PDF)

Table S2 Tumor growth inhibition rate of 9NC and 9NC-LP in HepG2 bearing nude mice (%).
(XLS)
