# Supplementary material for: Characterization of 9-Nitrocamptothecin Liposomes: Anticancer Properties and Mechanisms on Hepatocellular Carcinoma In Vitro and In Vivo
Source: PLoS One. 2011 Jun 9;6(6):e21064. doi: 10.1371/journal.pone.0021064 (PMC3111480; doi:10.1371/journal.pone.0021064)
Supplement: Table S1 — Effects of 9NC and 9NC-LP on the cell cycle progression. (PDF) [file pone.0021064.s001.pdf]

Table S1. Effects of 9NC and 9NC-LP on the cell cycle progression

Test compounds			HepG2				Bel-7402				Hep3B				L02			
Incubation time	Name	Concentration (μmol/L)	SubG1	G1	S	G2/M	SubG1	G1	S	G2/M	SubG1	G1	S	G2/M	SubG1	G1	S	G2/M
24h	9NC	Control	1.73±0.27	57.37±2.18	33.07±1.31	9.57±1.49	1.39±0.44	55.31±1.62	33.14±1.26	11.56±1.41	0.26±0.21	54.07±2.09	25.12±1.61	20.81±1.37	0.35±0.21	60.89±1.14	30.94±1.95	8.16±1.03
		0.01	2.80±0.42*	53.64±3.40	33.38±3.41	12.99±1.21*	2.18±0.69	51.93±2.45	34.38±2.26	13.68±1.72	0.32±0.17	57.30±2.66	24.95±1.55	17.75±2.02	0.33±0.19	44.22±2.01†	38.67±2.81*	17.11±1.06†
		0.025	6.73±0.49†	0.09±1.12†	55.21±3.72†	44.71±3.41†	5.62±0.39†	7.84±0.49†	43.46±2.17†	48.69±2.07†	0.35±0.23	56.84±2.32	24.62±1.36	18.54±1.92	0.31±0.22	24.25±1.94†	65.64±2.73†	10.11±1.33
		0.05	9.42±1.11†	0.00†	99.21±2.11†	0.78±0.68†	7.31±0.77†	4.79±0.33†	79.49±2.41†	15.72±1.12*	0.32±0.22	56.59±2.51	20.25±1.13*	23.16±1.91	0.24±0.14	15.33±1.84†	80.24±3.55†	4.43±0.52*
		0.1	13.38±1.68†	0.00†	100.00†	0.00†	9.87±0.57†	1.88±0.34†	96.76±2.38†	1.37±0.27†	1.10±0.27*	57.84±2.45	15.88±1.24†	26.28±1.41†	0.31±0.15	16.33±1.63†	83.67±2.65†	0.00†
		0.5	14.25±1.87†	0.00†	100.00†	0.00†	13.65±0.75†	0.00†	100.00†	0.00†	1.03±0.32*	42.99±2.93†	36.74±2.61†	20.27±2.21	0.35±0.11	0.00†	100.00†	0.00†
		1	24.62±1.83†	0.00†	100.00†	0.00†	19.23±1.34†	0.00†	100.00†	0.00†	3.05±0.39†	43.57±3.01†	54.18±2.21†	2.24±1.15†	9.39±0.43†	0.00†	100.00†	0.00†
	9NC-LP	Control	1.81±0.19	55.75±1.70	35.46±1.35	8.78±1.27	1.26±0.28	56.49±1.53	31.62±1.15	11.89±0.93	0.23±0.11	56.99±1.29	30.99±1.15	12.02±0.67	0.75±0.27	59.92±1.87	30.59±1.65	9.49±1.45
		0.01	2.21±0.30	50.22±3.59	36.97±2.33	12.81±2.41	2.09±0.56	55.83±2.26	31.74±2.21	12.44±1.78	2.62±0.13†	64.81±2.56*	29.36±2.17	5.83±0.74†	0.74±0.17	33.45±1.71†	46.84±1.84†	19.72±1.37†
		0.025	2.96±0.54	32.47±3.50†	41.41±2.49*	26.12±1.99†	3.71±0.65*	36.09±2.57†	39.57±2.31*	24.33±1.42†	2.61±0.24†	59.84±2.24	32.99±2.19	7.17±0.58†	0.82±0.26	26.19±0.82†	69.06±2.18†	4.75±0.55*
		0.05	3.43±0.51*	4.89±0.44†	62.83±4.08†	32.28±2.45†	3.88±0.36†	7.66±1.08†	59.24±2.65†	33.09±1.66†	3.23±0.18†	60.22±2.77	38.45±1.98†	1.33±0.24†	1.27±0.47	24.89±2.05†	75.11±2.39†	0.00†
		0.1	18.48±1.64†	34.80±3.62†	65.20±2.89†	0.00†	17.52±1.28†	3.88±0.69†	89.76±2.93†	6.37±1.04†	4.08±0.26†	58.16±2.37	41.22±2,31†	0.63±0.72†	1.25±0.86	3.75±0.97†	96.25±2.46†	0.00†
		0.5	21.92±2.13†	0.00†	100.00†	0.00†	20.19±1.16†	1.56±1.71†	98.43±2.84†	0.00†	4.19±0.18†	60.64±2.49	38.56±1.03†	0.80±0.61†	1.94±0.43*	16.46±0.77†	83.54±2.19†	0.00†
		1	40.67±2.47†	0.00†	100.00†	0.00†	34.78±1.57†	0.00†	100.00†	0.00†	5.04±0.22†	71.71±2.79†	26.81±2.54	1.48±0.78†	10.81±0.45†	57.39±2.22	42.61±1.87†	0.00†
48h	9NC	Control	1.66±0.33	58.36±3.29	31.05±1.88	10.59±1.29	1.55±0.29	60.47±1.68	28.38±1.43	11.16±1.33	1.69±0.23	68.89±1.71	21.7±1.31	9.41±1.61	1.43±0.32	68.1±2.34	19.89±1.67	12.01±1.26
		0.01	8.58±0.73†	43.77±3.56†	27.94±2.51	28.29±3.49†	6.74±1.26†	48.35±2.62†	26.83±2.57	24.81±1.61†	1.47±0.24	78.76±2.69†	16.44±1.84*	4.80±0.69*	5.32±0.66†	41.67±1.86†	19.3±2.20	39.03±1.49†
		0.025	9.28±1.22†	31.19±2.49†	26.83±3.09	41.98±3.57†	7.97±1.63†	38.22±2.58†	24.64±2.49†	37.15±2.18†	2.77±0.39*	82.09±2.75†	14.02±1.67†	3.89±0.63*	6.58±0.47†	21.93±1.68†	20.03±1.35	58.03±2.26†
		0.05	27.17±1.98†	22.63±2.89†	1.56±0.77†	75.81±2.34†	23.22±2.34†	28.51±1.61†	6.32±1.55†	65.17±3.03†	4.93±0.22†	68.14±3.35	21.82±1.90	10.05±0.37	12.16±0.83†	10.47±1.28†	44.99±2.81†	44.54±2.29†
		0.1	50.78±2.31†	13.56±0.72†	75.30±2.89†	11.14±0.68	42.85±2.31†	16.38±2.09†	58.85±2.05†	24.77±2.12†	4.16±0.26†	68.52±3.15	18.19±2.12	13.29±0.29*	19.15±1.01†	0.78±0.32†	90.74±3.00†	8.48±0.54*
		0.5	53.09±2.37†	0.00†	92.53±3.13†	7.47±0.82*	49.64±2.30†	1.06±0.44†	86.47±1.70†	12.48±1.88	4.51±0.37†	28.39±2.41†	39.29±2.34†	32.32±1.81†	14.37±1.09†	3.01±0.23†	91.63±3.64†	5.36±0.76†
		1	62.75±3.43†	0.00†	100.00†	0.00†	51.62±2.07†	0.00†	98.28±1.85†	1.71±0.36†	5.09±0.43†	28.74±2.90†	62.22±3.08†	9.04±0.24	30.39±1.12†	10.2±1.17†	81.88±3.07†	7.91±1.06*
	9NC-LP	Control	1.56±0.30	57.39±3.80	32.04±2.28	10.58±0.74	1.67±0.55	59.57±1.69	27.84±1.32	12.58±1.91	1.71±0.21	67.93±1.80	21.43±1.28	10.64±1.11	1.06±0.31	66.81±2.42	19.74±1.18	13.45±1.12
		0.01	8.44±0.69†	40.36±2.21†	30.88±3.83	28.76±0.98†	7.51±1.35†	40.84±2.59†	27.62±2.53	31.53±1.71†	2.04±0.32	67.14±2.87	22.18±1.55	10.68±2.21	5.91±0.29†	25.12±1.62†	23.81±1.32*	51.08±2.13†
		0.025	19.01±1.28†	24.65±2.26†	8.07±0.17†	67.28±3.99†	16.37±1.35†	29.04±2.61†	12.29±1.62†	58.68±2.71†	2.76±0.35*	32.13±2.13†	21.28±2.11	46.59±3.02†	7.58±0.64†	12.12±0.22†	22.72±1.87	65.16±2.98†
		0.05	45.51±2.49†	11.15±0.33†	1.94±0.27†	86.91±2.99†	39.24±2.38†	9.44±1.34†	6.83±1.65†	83.74±2.55†	3.55±0.36†	28.73±2.71†	45.39±3.18†	25.87±2.42†	8.42±0.66†	11.85±0.16†	13.95±0.17†	74.2±2.71†
		0.1	57.92±3.17†	8.39±1.86†	64.87±4.39†	26.74±2.26†	51.62±3.06†	0.49±0.32†	49.04±2.34†	50.48±2.75†	5.04±0.42†	43.82±2.72†	40.22±2.93†	15.96±1.53†	13.09±0.47†	5.03±0.33†	70.14±3.22†	24.83±2.26†
		0.5	64.78±3.28†	0.00†	92.17±5.01†	7.83±0.22*	59.57±2.39†	0.00†	78.66±2.32†	21.35±1.24†	5.66±0.43†	24.64±3.08†	43.35±3.61†	32.01±3.14†	17.51±0.72†	0.00†	94.35±3.96†	5.65±0.39†
		1	61.21±3.33†	0.00†	98.53±4.14†	1.47±1.20†	66.71±3.17†	0.00†	90.36±2.85†	9.64±1.84	5.49±0.47†	49.07±3.29†	46.15±2.85†	4.78±0.73†	37.56±0.76†	10.10±0.18†	87.97±3.6†	1.93±0.21†
72h	9NC	Control	1.78±0.73	65.20±1.94	23.55±1.88	11.25±2.32	1.82±0.46	67.67±2.17	22.31±1.34	10.03±1.62	2.09±0.37	71.31±1.25	21.00±1.08	7.69±1.70	1.43±0.45	69.35±2.37	21.33±1.74	9.33±1.63
		0.01	9.51±1.23†	58.61±3.71	22.80±2.28	18.59±2.22†	8.73±1.93†	63.37±2.81	21.62±2.17	15.02±2.21*	2.11±0.43	72.99±3.32	19.34±1.61	7.67±0.45	6.69±0.69†	56.31±2.67†	24.66±2.05	19.03±1.18†
		0.025	18.34±1.68†	32.37±2.94†	22.97±2.86	44.66±4.36†	15.47±1.36†	45.01±2.59†	22.51±2.03	32.49±2.67†	2.92±0.59	72.70±3.11	19.93±1.35	7.37±0.71	9.58±0.74†	22.03±2.14†	24.21±2.22	53.76±3.52†
		0.05	45.21±3.22†	3.93±1.23†	24.71±3.23	71.36±5.76†	29.54±3.14†	15.10±2.20†	21.16±2.53	63.73±2.49†	4.25±0.36†	60.04±3.74*	25.74±2.17*	14.23±1.38†	9.83±0.75†	9.75±1.32†	20.11±1.96	70.14±3.39†
		0.1	58.84±3.61†	0.19±0.96†	0.00†	99.81±4.87†	47.66±3.02†	3.23±1.11†	17.34±1.93*	79.44±3.32†	6.01±0.82†	67.04±3.03	20.53±2.03	12.43±1.12*	14.06±0.88†	5.72±0.87†	4.3±0.33†	89.98±4.68†
		0.5	64.91±3.98†	9.37±1.67†	13.78±2.14†	76.85±4.22†	59.39±3.49†	1.76±1.31†	0.00†	98.25±3.56†	6.03±1.37†	29.89±2.55†	31.51±2.97*	38.60±2.98†	21.97±0.74†	0.00†	83.76±3.48†	16.24±1.53†
		1	73.34±4.33†	4.38±1.42†	19.87±2.31	75.75±4.56†	63.27±2.97†	6.72±1.80†	5.56±1.28†	87.72±3.24†	15.53±1.53†	39.72±3.07†	36.72±2.84†	23.56±2.32†	33.57±0.63†	0.00†	76.34±3.47†	23.66±2.6†
	9NC-LP	Control	1.92±0.37	66.69±2.19	25.24±2.07	8.07±1.26	1.99±0.76	68.52±1.98	23.24±1.45	8.25±1.75	2.15±0.38	73.08±1.93	21.21±1.24	5.71±0.76	1.81±0.44	69.97±2.87	19.31±1.21	10.72±1.09
		0.01	10.22±2.47†	42.35±3.93†	25.47±3.71	32.17±2.78†	9.54±1.32†	56.05±2.75†	22.18±2.48	21.76±2.61†	2.85±0.68	75.12±2.54	18.97±1.37	5.91±1.42	5.56±0.89†	9.71±1.01†	2.17±0.36†	88.11±3.64†
		0.025	26.73±3.45†	17.82±2.19†	20.82±3.58	61.37±4.16†	24.49±2.90†	34.09±2.37†	16.57±2.11*	49.35±2.93†	3.03±0.77	73.70±3.56	22.05±2.21	4.25±1.17	8.29±0.69†	4.75±1.02†	0.33±0.28†	94.92±3.24†
		0.05	57.59±4.89†	3.04±1.27†	1.65±1.29†	95.31±4.65†	49.17±3.21†	16.45±2.16†	7.12±2.09†	76.43±3.64†	5.05±0.71†	70.25±3.55	20.15±2.38	9.60±1.27*	10.31±0.69†	0.32±0.19†	1.32±0.36†	98.36±4.38†
		0.1	66.48±3.23†	1.46±0.56†	3.33±0.17†	95.21±3.76†	57.32±3.08†	7.12±0.87†	0.13±0.33†	92.76±3.55†	6.91±0.83†	66.69±3.81	20.61±3.20	12.70±2.93*	16.18±0.84†	5.40±0.83†	7.4±1.24†	87.2±3.74†
		0.5	74.77±4.31†	3.67±0.77†	14.81±1.32†	81.52±3.89†	63.14±3.81†	0.00†	0.60±0.35†	99.39±3.12†	7.67±1.75†	24.49±3.94†	39.33±3.10†	36.17±3.21†	22.86±0.72†	0.00†	29.35±2.52†	70.65±4.22†
		1	70.46±3.82†	0.04±0.32†	20.72±4.27	79.24±2.97†	65.83±2.72†	0.38±0.47†	10.52±2.74†	89.10±3.03†	18.28±1.48†	39.4±3.06†	42.06±2.62†	18.54±3.34†	35.45±0.74†	0.00†	50.39±3.58†	49.61±2.84†

Cell treated with 4‰DMSO as control in 9NC group, Cell treated with Liposomes (free) as control in 9NC-LP group. *$P < 0.05$, †$P < 0.01$ compared with the controls.
